# EXPLORING THE CAREGIVING CAREER: VARIATIONS IN SPOUSES’ CONCERNS

**DOI:** 10.1093/geroni/igz038.2299

**Published:** 2019-11-08

**Authors:** Marie Y Savundranayagam, Kaitlyn Terrana

**Affiliations:** Western University, London, Ontario, Canada

## Abstract

Caregiver identity theory posits that family caregivers’ relationship identity changes across five phases of the caregiving career. As the career unfolds, the relationship identity changes from one comprised primarily of the spousal role (phase 1) to one comprised equally by spousal and caregiver roles (phase 3), and one comprised primarily by the caregiver role (phase 5). This study investigated whether spouses/partners’ most important concerns about caregiving varied across the caregiving career. Participants included 135 caregivers of spouses/partners with a chronic condition. They were asked to identify their most important concern related to caregiving, along with demographic questions. Thematic analyses of their concerns yielded eight themes focused on the caregiver or dyad. Caregiver focused themes included burden, providing best possible care, worry about ability to care, physical health, financial concerns, and needing/managing help. Dyadic themes were communication and relational deprivation. Differences across the caregiving career were found in terms of ranked proportion of concerns. The most common concerns in phase 1 were equally distributed across communication, needing/managing help, and providing best possible care. Phase 2’s most common concern was providing the best possible care. Phase 3’s most common concern was worry about ability to care. The most common concerns in phase 4 were equally distributed across burden and worry about ability to care. Phase 5’s most common concern was burden. Findings reveal there are differential concerns across the caregiving career that align with a greater focus on relational factors earlier in the career and caregiver burden later in the career.

